# A review of technical and social methods for observing and monitoring extreme rainfall events

**DOI:** 10.4102/jamba.v17i1.1907

**Published:** 2025-09-20

**Authors:** Simangaliso I. Mnyandu, Ntombifuthi P. Nzimande

**Affiliations:** 1 Department of Geography, College of Humanities, University of KwaZulu-Natal, Durban, South Africa

**Keywords:** extreme rainfall events, indigenous knowledge systems, rainfall observation, integrated approaches, climate risk management

## Abstract

Recent extreme weather events driven by climatic variation and change have severely impacted infrastructure, human life, and ecosystems globally, increasing the urgency for Early Warning Systems (EWS) and hybrid methods to observe extreme rainfall events. This article reviews existing studies on technical and social methods for monitoring extreme rainfall to explore the integration of these approaches in rainfall observation. Given the limitations of technical and social methods when used in isolation, it has become evident that hybrid approaches that integrate social and technical methods could enhance preparedness for catastrophic rainfall events. Therefore, the Preferred Reporting Items for Systematic Reviews and Meta-Analyses method was employed to identify and analyse studies on rainfall observation. A total of 3786 articles were identified from different databases, and after applying various filters, 144 studies were selected and analysed to improve our understanding of rainfall monitoring approaches. The key findings of this review indicate four main trends: (1) technical methods dominate over social methods because of their perceived reliability and precision; (2) in the Global North, only technical methods are used, with no recorded studies incorporating social approaches; (3) social methods, along with integrated approaches, are more prevalent in the Global South; and (4) integrated methods remain limited despite their potential to enhance rainfall monitoring. This review provides insights into the benefits of combining technical and social methods, highlighting their complementary strengths. By bridging the gap between high-tech solutions and community-based monitoring, integrating these approaches could improve EWS and strengthen climate resilience.

**Contribution:**

This review addresses the gap in integrating technical and social methods for rainfall monitoring, emphasising their complementary strengths. It highlights the limited use of integrated approaches, particularly in the Global North, despite their potential to enhance EWS. This research advocates for inclusive and effective climate risk management by bridging high-tech solutions with community-based monitoring.

## Introduction

Advancements in meteorological science over the past century have significantly improved rainfall monitoring and observation methods, crucial for understanding climate change (Allan et al. 2023). Extreme rainfall leads to floods, landslides and severe infrastructure damage, accounting for approximately 55% of economic losses and 38% of disaster-related deaths globally (Marengo et al. 2023). Over the last 35 years, the frequency of these events has increased and they are closely linked to climate change (Hait & Sahu 2024). Climate change has caused a 90% rise in natural hazards associated with extreme weather, resulting in 40 000 to 50 000 annual deaths, disproportionately affecting the poorest populations (Marengo et al. 2023; Ritchie, Rosado & Roser 2024). In 2024, extreme rainfall affected many regions worldwide (Rawat et al. 2024), underscoring the need for enhanced rainfall monitoring and adaptive strategies to mitigate these extreme weather effects globally (Vincent 2024). These strategies would not only help in mitigating the immediate impacts of extreme rainfall events but would also support long-term planning for sustainable urban development and climate adaptation, especially as the intensity of these events continues to increase drastically.

The increased frequency and intensity of extreme rainfall events highlight an urgent global challenge that intersects directly with the Sustainable Development Goals (SDGs), particularly SDG 11 (Sustainable Cities and Communities) and SDG 13 (Climate Action), among others. As severe weather events increase, there is a growing need for resilient infrastructure and disaster preparedness to mitigate damage, as these events disproportionately impact vulnerable populations and hinder sustainable development (Hsieh & Yeh 2024). Several international policies, including the Sendai Framework for Disaster Risk Reduction (2015–2030) and the Paris Agreement, have been developed to address these challenges over the years (Carrington, Ranse & Hammad 2021). These initiatives emphasise resilience building, Early Warning Systems (EWS) and climate adaptation. The Intergovernmental Panel on Climate Change (IPCC) also highlights the need for enhanced rainfall monitoring and adaptive measures (Allan et al. 2023). This underscores the global need for improved observation and EWS.

Historically, observation of extreme rainfall began with simple instruments such as rain gauges, which provided fundamental yet essential data on rainfall events (Dube et al. 2023). Over time, increasingly advanced instruments and techniques in meteorology have been developed. Major advancements in radar and satellite technology have enabled real-time rainfall monitoring, providing a deeper understanding of atmospheric dynamics (Liu et al. 2021). In recent years, rainfall observation has seen a noticeable evolution in data collection methodologies and the integration of sophisticated computational models and data assimilation techniques (Biondi et al. 2024). Advanced algorithms and high-performance computing have altered how meteorologists examine and interpret observational data for forecasting (Biondi et al. 2024). The computing systems are now responsible for making predictions with little human intervention and consideration for local variations. Modern observational devices, such as weather drones, have further expanded the systems’ capabilities for weather monitoring (Bansod et al. 2017). These tools also provide high-resolution, real-time data from previously inaccessible areas and enhance understanding of microclimates (Bansod et al. 2017).

Advanced monitoring techniques, such as satellite observations, rain gauge sensors and sophisticated modelling, are crucial for mitigating the effects of severe rainfall events by providing early warnings that enable effective community response (Murphy 2014). However, accessibility and user-friendliness remain significant barriers because of costs and access to specialised knowledge. Existing methods for monitoring rainfall face limitations that reduce their effectiveness in addressing localised or unexpected extreme events (Segoni, Piciullo & Gariano 2018). These limitations include insufficient community involvement, inadequate data capture and an over-reliance on top-down, heavy technology solutions. Hybrid methods are needed to address extreme weather response challenges by incorporating community-based approaches. These approaches prioritise active community participation, integrating locally devised strategies and indigenous knowledge system (IKS) into data collection, decision-making and disaster management (Murphy 2014).

There is still a significant technological bias as some countries and communities find it difficult to afford such equipment because of its high cost (Sufri et al. 2020). Innovative and hybrid methods must be developed to bridge the gap of local variations by using community science and crowd-sourcing to observe and monitor extreme rainfall events (Medina et al. 2024). While advanced technology may be effective for many countries, it is often inaccessible to low-income communities, making local methods a viable alternative (Nsabagwa et al. 2019). Thus, this review aims to conduct a methodological review to explore the extent to which past methodologies integrate community knowledge. Furthermore, this review will reveal research and methodological gaps for further research.

## Research methods and design

### Literature search

This review examines methods for observing rainfall globally, acknowledging variations in rainfall patterns and community engagement across different geographical areas, particularly between the Global North and Global South. The Preferred Reporting Items for Systematic Reviews and Meta-Analyses (PRISMA) method was applied as it is an internationally recognised and widely accepted review methodology. Three electronic databases were accessed to search for literature on the topic reviewed in this article: Google Scholar, Scopus and Web of Science. The search terms were separated into two parts: technological and social methods. Technological methods refer to instruments and technologies used to measure rainfall, while social methods include community and local knowledge for observing rainfall. The search terms used were: ‘rainfall monitoring’ OR ‘rainfall observations’, ‘weather observations’ AND ‘rain gauges’, ‘radar rainfall’ OR ‘satellite rainfall’ OR ‘remote sensing of rainfall’, ‘rainfall sensors’ AND ‘weather stations’, ‘LIDAR for precipitation’ AND ‘participatory rainfall monitoring’, ‘citizen science’ OR ‘crowd-sourcing rainfall’ and ‘indigenous rainfall knowledge’ OR ‘indigenous methods of observing rainfall’ OR ‘local methods for observing rainfall’.

### Selection criteria

The review focused on English-language articles published between January 2004 and December 2024, covering technical and community-based rainfall monitoring, observation and disaster preparedness. The 20-year period was selected to capture methodological advances since the early 2000s, particularly the shifts from traditional instruments to satellite technology for more precise data with free access (Hemati et al. 2021). Eligible studies offered empirical data or theoretical insights on the effectiveness of rainfall monitoring methods, with a global scope to reflect diverse practices. Only peer-reviewed articles with case studies were included, while older, non-peer-reviewed, grey literature and studies unrelated to rainfall monitoring were excluded.

### Overall preferred reporting items for systematic reviews and meta-analyses search

**FIGURE 1:**
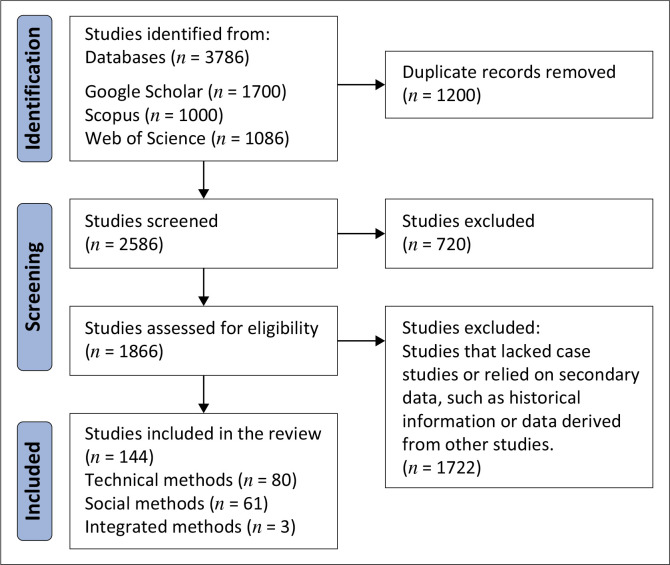
Flow diagram of the selection process based on preferred reporting items for systematic reviews and meta-analyses.

The review process focused on case study-based methodologies, excluding studies that rely on historical data. During the identification phase, 3786 studies were retrieved from the databases. After removing 1200 duplicates, 2586 studies were screened for relevance. In the screening phase, 720 records were excluded for not meeting the initial criteria, leaving 1866 studies to be assessed for eligibility. Of these, 1722 were excluded because they either lacked case studies or relied on secondary data, such as historical information or data derived from other studies. Ultimately, 144 studies were included in the review, categorised into 80 on technical, 61 on social and 3 on integrated methods (Figure 1). The smaller number of studies that used the integrated method highlights the limitation in research that combines technical and social approaches, suggesting a possible area for further exploration.

### Ethical considerations

Ethical approval to conduct this study was obtained from the University of KwaZulu-Natal Research Ethics Committee (No. 00021368).

## Results

### Publications trends

**FIGURE 2:**
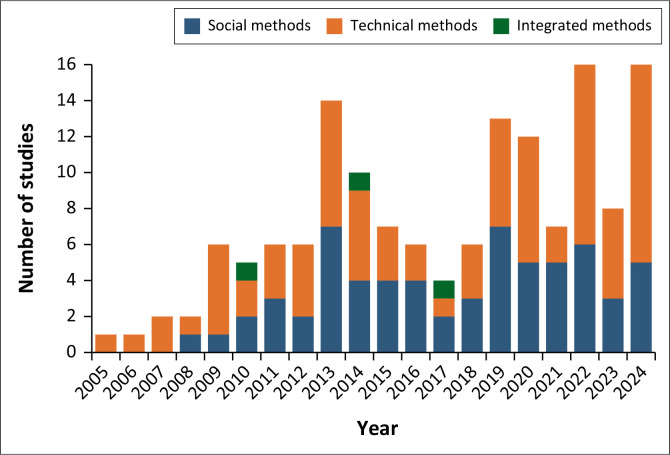
Trends of publications in rainfall observation methods.

Between 2004 and 2009, the adoption of social methods was minimal, with little to no studies conducted, while technical methods consistently occurred frequently (Figure 2). This reflects a heavy reliance on scientific instruments for rainfall monitoring, with little use of social methods. During this period, remote sensing data also began to gain traction as it became increasingly utilised and accessible as open-source data (Hemati et al. 2021). From 2010 to 2012, social studies methods started gaining prominence, although still lagging behind technical methods. Social methods studies ranged from 0 to 2, while technical methods fluctuated between 1 and 4 studies maintaining dominance. A significant shift occurred from 2013 to 2016, when social methods grew rapidly, peaking at 8 studies in 2013. This spike marked the first instance where social methods outpaced technical methods, suggesting increased recognition or application of social methods in rainfall monitoring. Between 2017 and 2024, social methods studies fluctuated, oscillating between 2 and 8. The studies that employed integrated approaches were limited and sporadic, with only one study identified yearly in 2010, 2014 and 2017.

### Geographical distribution of the selected publications

**FIGURE 3:**
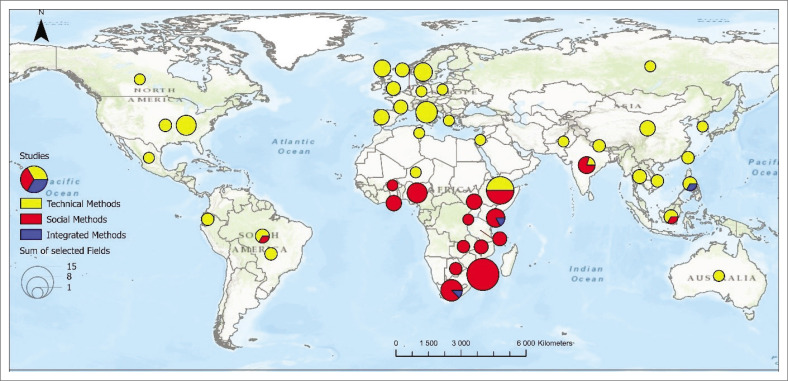
Geographical distribution of the selected studies on a global scale.

The distribution of studies on rainfall observation reveals a notable disparity in methodological approaches globally. Figure 3 shows that the studies employing technical methods are globally dispersed, in Europe, Asia, North and South America, and Africa. However, most studies that used technical methods are primarily found in the Global North when comparing these two hemispheres. There are studies that used technical methods in the Global South, but they are far fewer in number. There is an uneven and sparse distribution of studies, with most studies conducted in the United States, North America, the United Kingdom and Italy. At the same time, the rest of the regions have one to two studies each.

In contrast, studies employing social methods are wholly in the Global South and heavily concentrated in Africa, with Zimbabwe standing out significantly with a record of 15 studies. Other countries with a considerable number of studies include Uganda, Ethiopia, South Africa, Kenya and Tanzania, while the rest have only one or two studies each. Social methods are minimally represented outside Africa, with only three recorded studies conducted in other Global South countries such as South America, India and Mexico. There are no studies that have been documented in the Global North employing social methods on extreme rainfall observation and monitoring, highlighting a significant gap in research on these methods beyond Africa. The regional disparity in social methodologies highlights an imbalance, emphasising the need for further research in areas where these methods are largely absent.

The integrated approach was used in only three studies from the Global South, which include South Africa, Kenya and the Philippines. A country can use both methods in different studies, with one study focusing exclusively on the social method and another on the technical method. These methods are applied separately, without integration. For example, countries such as South America, Ethiopia, India and Indonesia are represented with a pie chart on the map, using two colours (red and yellow) to show that both methods have been used. The results show little discussion or adoption of the integrated methods. This lack of integration could stem from several factors, including institutional biases favouring scientific approaches (Glazebrook 2021), limited documentation or recognition of social methods as a credible source of knowledge and gaps in collaborative frameworks that bring scientific and local communities together (Balogun & Kalusopa 2021).

### Methodologies in rainfall monitoring

**FIGURE 4:**
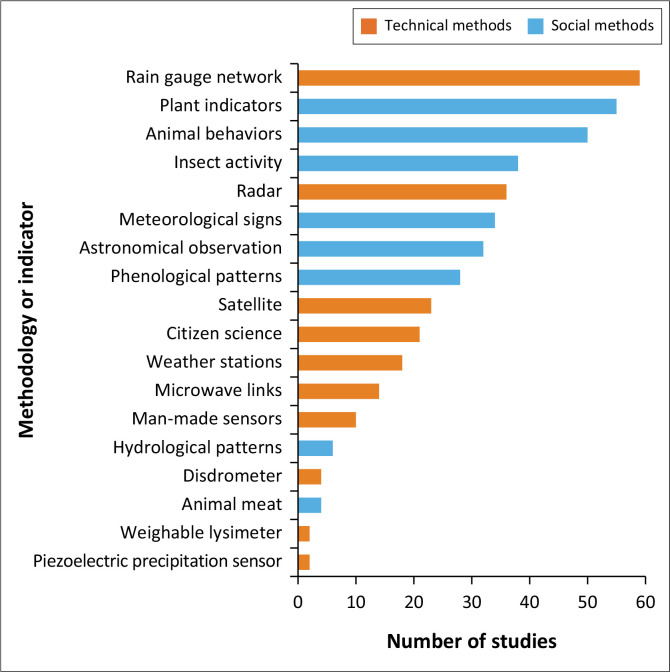
Overview of rainfall observation methodologies.

The reviewed publications provided insight into the distribution of methodologies used in rainfall monitoring, with an analysis of their frequency revealing significant trends and insights into their application. Figure 4 shows that the highest number of studies using the technical method, 59 studies, used the rain gauge network. Its widespread use reflects its reliability, cost-effectiveness and ability to provide direct precipitation measurements (Buytaert et al. 2006; Chen, Wei & Yeh 2007). This is followed by radar, with 36 studies, and satellite technologies, with 23 studies. Both radar and satellite systems demonstrate the increasing reliance on advanced technological methods for broader spatial coverage and real-time data acquisition, which are crucial for large-scale weather monitoring and hydrological forecasting (Wetchayont et al. 2023).

Social methods are rooted in observations, such as plant indicators in 55 studies, animal behaviours in 50 studies and insect activity in 38 studies, and feature prominently in rainfall prediction methodologies. Such indicators often reflect environmental changes indirectly, providing valuable insights into the localised impacts of rainfall and weather patterns (Balehegn et al. 2019; Shoko & Shoko 2013). Other methods include meteorological signs with 34 studies, astronomical observations with 32 studies and phenological patterns with 28 studies. These methods underscore blending traditional wisdom, such as observing celestial events and environmental cues, with structured scientific methodologies (Nyadzi et al. 2020). Their relatively high research representation demonstrates their continued relevance in community-based rainfall prediction, especially in rural and underserved areas. Emerging technologies such as citizen science, with 21 studies; microwave links, with 14 studies; and man-made sensors, with 10 studies, illustrate the growing trend towards participatory and innovative approaches.

Niche technologies, such as the disdrometer with four studies, the weighable lysimeter with two studies, and the piezoelectric precipitation sensor with two studies, highlight experimental and specialised tools often in developmental phases or suited to specific contexts. In addition, less frequently studied approaches such as animal meat indicators with four studies and hydrological patterns with six studies may be context-specific or face constraints related to accuracy, practicality or the availability of alternative methods. The data suggest a diverse methodological landscape where technical methods dominate because of their reliability and widespread applicability. However, the prominence of indicators signals the importance of traditional knowledge, particularly in contexts where technology is inaccessible (Fabiyi & Oloukoi 2013). Emerging technologies and participatory approaches such as citizen science and crowd-sourced methods reflect innovation and inclusivity in environmental monitoring (O’Hara et al. 2023).

## Discussion

In recent years, technological advancements have significantly improved rainfall observation methods, enhancing extreme rainfall monitoring and prediction capabilities. While modern techniques have introduced more precise and data-driven approaches, traditional methods play a crucial role in rainfall observation, particularly in certain communities (Subramanian & Pisupati 2010). This review explores two primary categories of rainfall monitoring methods, technical and social, and their potential integration. Each method offers distinct advantages and limitations, influencing the accuracy and reliability of rainfall observation, monitoring and predicting extreme rainfall events. In Figure 2, the study’s findings indicated few studies from early 2004 to 2008, indicating a possible lack of research on rainfall observation methodologies and extreme rainfall events. This could be attributed to the data availability of rainfall events and the shift in research priorities towards other environmental hazards and disasters, such as droughts, wildfires and climate change adaptation techniques (Singh & Smithers 2020; Smithers et al. 2001). However, this changed as a noticeable rise in research on observation methods and extreme rainfall events emerged from 2009 to 2024. This increase suggests that more extreme rainfall events were occurring, causing significant damage to society and thus sparking research interests from scholars. This growing academic focus reflects the urgency of understanding extreme weather patterns and aligns with broader global efforts to combat climate change. Moreover, the growing body of research may also be linked to the progress in addressing climate change through the SDGs, as the adoption of these goals in 2015 sparked increased awareness and action, particularly regarding climate-related risks, which led to a surge in related research (Li et al. 2022). As the SDGs gained momentum, so did the urgency for understanding and mitigating the effects of extreme weather events. The SDGs established a direct link between climate change and its associated impacts, prompting the development of several climate change policies, summits and conferences.

The findings of Figure 1 and Figure 2 illustrated that the majority of the studies used technical rainfall observation methods, with many of these employed in the Global North. This preference for technical methods may be attributed to their perceived reliability, accuracy, advanced technology and infrastructure availability in these regions. For example, Riffel (2020) highlighted the disparity in adopting technical observation methods between the Global North and Global South. This disparity can be attributed to several factors, including the higher accuracy of technical observation methods compared to social methods. Reasons for the predominant adoption of technical observation methods is also supported by Mishra (2013), who postulated that rain gauges and their density play a critical role in the accuracy of rainfall measurements during their study in India. Rossi et al. (2017) asserted that rain gauges are more accurate than other methods and are even used to correct errors in satellite and radar data. These findings highlight why technical methods are favoured by most countries and studies, which concurs with the findings of this study’s observations on the reliance on technical methods for improved rainfall monitoring accuracy.

Furthermore, the widespread use of technical methods in the Global North is also largely common because of the availability of simple, low-cost instruments such as rain gauges and weather stations, which are affordable and accessible to communities, even in areas with limited resources (Davids et al. 2019; Paul et al. 2020). Despite technical methods being utilised more in the Global North, researchers in the Global South have also adopted these methods because of the availability of low-cost instruments, such as rain gauges, which are affordable and accessible to communities. Davids et al. (2019) conducted a study in Nepal that involved training local community members to construct, install and maintain low-cost soda bottle rain gauges. These gauges were simple to assemble using readily available materials, enabling individuals with minimal technical skills to participate in rainfall monitoring. This citizen science initiative successfully collected rainfall data in remote areas, demonstrating that rain gauges and citizen science are valuable tools in underserved communities with limited access to complex meteorological equipment. In addition, O’Hara et al. (2023) and Medina et al. (2024) demonstrated that citizen science and crowdsourcing initiatives using rain gauges and simple weather stations were highly effective. These initiatives provided more localised rainfall data, addressed coverage gaps and improved the accuracy of weather predictions. By leveraging community participation, these projects enhanced the spatial coverage of rainfall data collection, making it possible to capture rainfall variations that may otherwise go unnoticed in formal meteorological networks.

Despite the published success of these methods, limitations also exist in that rain gauges and weather stations require a high density in a given area to collect accurate data (Mishra 2013). For highly accurate results, Wood, Jones and Moore (2000) suggested that rain gauges should ideally be spaced at a density of 1 gauge per 10 square kilometres (10 000 000 square metres) or higher for larger-scale hydrological events. These studies indicated that when the density of rain gauges was lower than this threshold, the accuracy of rainfall measurements was compromised, leading to potential underestimation or overestimation of precipitation levels, which in turn affected hydrological modelling and EWS. Similarly, Vieux and Vieux (2005) asserted that for an area of 100 km^2^, at least three rain gauges would be necessary, resulting in a density of one gauge per 33.3 km^2^. Meeting these density requirements is often challenging, particularly in remote or resource-constrained areas, leading to lower accuracy and unreliable estimations (Mishra 2013). In addition, although community-based environmental monitoring has advantages, these methods require proper maintenance and calibration to avoid inaccurate readings, especially in crowdsourced and citizen science initiatives (Chen, Behl & Goodall 2021). This was the case in the study conducted by Jacobs (2016), which found that despite training community members, manual errors and inadequate maintenance can negatively impact data quality, potentially reducing prediction accuracy. Jacobs (2016) identified several challenges associated with crowdsourced data, including varying observer expertise, inconsistent data collection methods and potential biases in data identification. These challenges highlight the importance of ongoing training, support and periodic validation to ensure the reliability of community-collected rainfall data.

In a bid to address these limitation, advanced technologies, such as remote sensing tools, have been used to track rainfall movements, anticipate their impacts and provide valuable insights that some technical methods may not fully achieve. For example, Bournas and Baltas (2024), among others, successfully monitored rainfall events such as storms, determining their intensity, duration, scale and impacts. The emphasis was on the critical role of time in assessments, with studies highlighting the preference for technical methods because of their ability to provide timely, efficient and precise estimations (Ali, Deranadyan & Hairuly Umam 2020). Moreover, technical methods enable forecasting several days before rainfall events, enhancing preparedness and risk reduction (Tran et al. 2024). Some methods, such as satellite-derived rainfall estimates, can provide real-time monitoring and forecasting capabilities. Rojas et al. (2005) used satellite and ground-based observations in Eastern Africa to monitor real-time rainfall distribution. This improved early food security warnings by capturing rainfall variability and underscored the importance of reliable rainfall monitoring for agricultural planning and mitigating climate-related risks. Thus, as these advanced technologies can cover large areas, they provide valuable information in regions with limited ground-based monitoring infrastructure, such as in mountainous and arid areas of East Africa (Dinku et al. 2011). This shows that remote sensing tools are crucial in bridging observational gaps, particularly in data-scarce regions where traditional rain gauge networks are insufficient. Furthermore, integrating satellite-based measurements with *in situ* data has been widely recognised as a way to improve rainfall estimates and support EWS (Yu et al. 2020).

However, advanced methods are not without their challenges, as Dinku et al. (2011, 2013) highlighted that spatial variability in mountainous regions, infrequent rainfall in arid areas, cloud interference during storms, sparse rain gauge networks and coarse spatial resolutions hinder the accuracy of satellite rainfall estimation. In addition, the high cost of advanced methods limits accessibility for these methods in the Global South countries; although some data sources are open-access, those with richer and more accurate datasets are often costly (Sweeting 2018). Particularly, the high cost is often a result of the over-reliance of researchers in Africa on remote sensing tools from the Global North, as these systems are not only expensive but also come with limitations such as lower spatial and temporal resolution, data gaps and restricted integration with advanced modelling techniques (Centenaro et al. 2021). This reliance complicates data access and application, as African countries have limited control over the satellites, with only 35% of active satellites being utilised, none of which they have full authority over (Nakalembe, Devereux & Ginsburg 2024). These factors can negatively impact rainfall monitoring accuracy and the effectiveness of EWS and disaster preparedness. Despite these challenges, some studies have successfully utilised open-source data to meet their objectives. For instance, Mwange, Mulaku and Siriba (2016) examined spatial rainfall trends across Africa using open-source data, demonstrating the potential to achieve meaningful insights even with resource limitations. This highlights the importance of leveraging both traditional and advanced methods to enhance climate resilience in the Global South.

Social methods, also referred to as traditional indicators, are widely employed by communities and individuals to predict and observe rainfall. These methods rely on various atmospheric, biological and hydrological indicators, including cloud formations, wind patterns, animal behaviour and plant growth (Chanza & Musakwa 2022; Nkuba et al. 2020). Despite their longstanding role in community-based weather prediction, social methods are often underutilised and less studied compared to technical methods because of a perceived lack of scientific rigour and difficulties in standardising these practices across diverse regions and cultures (Irumva, Twagirayezu & Nizeyimana 2021). Nevertheless, social methods remain highly significant, particularly in the Global South, where rural communities often lack access to high-tech rainfall observation tools. For instance, a study conducted by Kom et al. (2022) revealed that farmers in South Africa employed several indigenous indicators to predict rainfall and other weather events. These indicators included observing animal behaviour, bird migration patterns, plant flowering or fruiting cycles and cloud formation. Farmers were better prepared for planting seasons using these methods and could adapt to potential droughts or floods. These indicators are deeply embedded in local knowledge systems passed down through generations, making them well-adapted to local environmental conditions and reflective of regional climate patterns (Kom et al. 2022).

Ayal et al. (2015) highlighted the historical marginalisation of social methods, as some scholars neither acknowledged nor incorporated them into their research because of the dominance of modern scientific approaches. This marginalisation is evident in Figure 2, which shows that social methods received little scholarly attention between 2004 and 2008. However, after 2009, there was a noticeable increase in recognition, coinciding with growing advocacy for the documentation of traditional knowledge (Hiwasaki et al. 2014). This shift reflects a broader effort to integrate indigenous and community-based knowledge into climate studies. Social methods, particularly in African communities, have long played a crucial role in rainfall prediction, drawing from cultural, historical and environmental practices (Basdew, Jiri & Mafongoya 2017). These traditional systems, adapted over centuries, continue to play an essential role in local climate resilience and adaptation efforts. Kabore et al. (2023) conducted a study in North Central Burkina Faso that identified various indigenous indicators farmers use to predict seasonal rainfall. This included animal behaviour, such as ants moving to higher ground or birds migrating unusually, as signs of impending rain. Farmers also observed changes in livestock meat, noting that higher fat content and certain textures indicated an abundant rainy season, while leaner meat suggested potential drought. These traditional methods passed down through generations, were crucial for farmers in regions with limited meteorological services, helping them adapt to climate variability and enhance resilience. While these traditional approaches hold significant influence within local contexts, they remain underutilised on a broader scale, especially outside Africa (Basdew et al. 2017).

Although indigenous and local knowledge have gained increased recognition in the Global North, especially in the context of climate change (Madzivhandila 2024; Shava et al. 2009), there remains a significant gap in research on using social methods for observing extreme rainfall events. In this article, we attempt to expose this gap, as no published peer-reviewed studies have focused on social methods for rainfall observation in the Global North. While some social studies in the Global North examine other climate-related events, none specifically focus on rainfall observation. The lack of adoption in the Global North primarily stems from a reliance on advanced meteorological technologies, which are perceived as more accurate and reliable. In addition, there may be limited research interest in social tools for rainfall observation, possibly because of the dominance of modern scientific paradigms that prioritise quantitative data over qualitative insights. This perception has contributed to the undervaluation of traditional methods and indigenous knowledge, further reinforcing the preference for high-tech solutions over community-based approaches (Hiwasaki et al. 2014).

The Global North is often associated with modernity and technological advancement, leading to cultural biases that view traditional social methods as outdated or unreliable (Riffel 2020). This cultural indifference has further limited the integration of social methods into formal meteorological practices, reinforcing the dominance of technology-driven approaches. However, it is also important to note that the data collected through social methods faces several inherent challenges. Ayal et al. (2015) highlight subjectivity as a significant issue, as individual observations can vary, leading to inconsistencies in recorded data. Alemayehu and Hizkeal (2022) further point out that social methods are often passed down orally, increasing the risk of knowledge loss or miscommunication over time. For instance, some participants in their study had forgotten certain information because of a lack of use over extended periods. Scientific validation of social methods, particularly in rainfall monitoring, is also challenging because of the absence of empirical benchmarks for comparison with modern instruments (Ankrah, Kwapong & Boateng 2021). While many studies have documented social methods, validation remains limited because of epistemological differences and the difficulties in standardising local knowledge (Irumva et al. 2021). Most existing research is small-scale or interdisciplinary rather than fully standardised experiments, further complicating integration with scientific approaches. These challenges underscore the need for further research to bridge these gaps and develop hybrid models incorporating social methods into scientific rainfall monitoring systems (Ebhuoma 2020; Nyadzi et al. 2022).

The other key finding of this review is that technical and social methods are often studied in isolation, with only three studies explicitly integrating the two approaches: Kipkorir, Mugalavai and Songok (2010), Hiwasaki et al. (2014) and Basdew et al. (2017). All three studies successfully integrated technical and social methods to monitor and observe rainfall. However, a notable critique is that data collection was conducted separately, with social and technical data gathered independently and integrated only during the analysis phase. Ebhuoma (2020) recommended simultaneous data collection and analysis within an integrated framework to achieve a more robust and holistic approach. Although other studies, such as Orlove et al. (2009), Ebhuoma (2020), Irumva et al. (2021) and Nyadzi et al. (2022), have discussed the importance of merging technical and social methods, they primarily explored methodologies for integration without fully implementing them. Interestingly, all three studies that employed integration originated in the Global South, with no documented cases from the Global North. This discrepancy aligns with the findings of this review, which indicate that the Global North heavily emphasises scientific observation because of historical biases, limited trust in social methods and the dominance of quantitative methodologies.

The potential benefits of an integrated approach are significant. Ebhuoma (2020) believes that merging technical and social methods can provide richer, more localised insights into rainfall variability and enhance the accuracy and applicability of EWS. For instance, while technical methods offer real-time, spatially precise data through tools such as rain gauges, remote sensing and radar, social methods can fill critical gaps, especially in informal or remote areas where infrastructure is limited, using social indicators such as plants and animals to predict extreme rainfall events. When these community findings are reported, they can be quantified and validated, allowing them to be incorporated into rainfall prediction models. Alternatively, forecasting modules can be designed to explicitly integrate local and indigenous knowledge, creating a hybrid approach that enhances both the spatial coverage and cultural relevance of rainfall monitoring efforts.

However, significant challenges remain. One critical issue that Basdew et al. (2017) identified is the fundamental difference in worldviews between the two approaches. Scientific methods prioritise standardised, universally applicable measurements, while social methods are more fluid, localised and context-specific (Nyadzi et al. 2020). This contrast can create tension when attempting to harmonise both knowledge systems, as each relies on distinct ways of interpreting and understanding rainfall events. Social methods tend to be holistic, experiential and rooted in cultural traditions, whereas scientific methods are highly quantifiable and driven by empirical validation (Ebhuoma & Simatele 2017). These differences can lead to misunderstandings or the undervaluation of social methods within the scientific community. More interdisciplinary research and participatory frameworks are necessary to bridge these divides, emphasising mutual respect and knowledge exchange between traditional and scientific perspectives (Kalanda-Joshua et al. 2011).

## Conclusion

Extreme rainfall and its environmental, social and economic impacts underscore the urgent need for improved monitoring to strengthen EWS and disaster preparedness. Traditionally, rainfall observation methods have operated independently, often proving ineffective in many countries and communities. Therefore, a more holistic approach that integrates social and scientific methods is necessary to maximise their combined potential. Such integration shows particular promise for marginalised communities that rely on limited or single-method systems. This review highlights the critical gap in current research, with very few studies adopting integrated approaches despite their clear potential to enhance monitoring frameworks. It emphasises the development of hybrid methodologies that combine localised, experiential knowledge with advanced scientific tools, recommending further research and advocating for stronger collaboration between scientists and local communities. This collaboration should prioritise co-creating knowledge by leveraging the complementary strengths of scientific expertise and community-driven practices.

Operationalising this integration can involve participatory data collection, community validation of technical outputs, use of homemade tools for local engagement and collaborative analysis among diverse stakeholders. To assess the effectiveness of such approaches, key indicators include the level of community involvement, alignment between social and technical data, usability of information for local decision-making, the presence of feedback mechanisms and evidence of policy uptake. These efforts aim to foster culturally relevant, inclusive and practical solutions that strengthen rainfall monitoring and resilience to extreme weather events. The successful development of integration methods depends on several conditions, including strong community engagement and trust, which enable local knowledge holders to collaborate actively with scientists. Capacity-building initiatives and workshops are also essential to equip technical experts and community members with the skills required for meaningful partnership. This study provides a unique analysis of technical and social rainfall observation methods, focusing on their integration. Unlike previous research that often examined these methods separately, this review highlights regional differences in their use, where technical methods dominate in the Global North. At the same time, social and integrated approaches are more common in the Global South. The findings reveal growing interest in hybrid methods and their potential to improve EWS and climate resilience.

By showcasing the strengths of both approaches, this research lays the foundation for more comprehensive and interdisciplinary rainfall monitoring strategies. It bridges the gap between technical hydrology and social sciences, underscoring the value of combining scientific precision with community insights to create more accurate and context-sensitive monitoring systems. The study calls on policymakers and researchers to adopt inclusive strategies integrating technological innovation with local knowledge, thereby advancing climate risk management and resilience. Future research should focus on developing and testing integrated rainfall monitoring frameworks that blend community-based observations with scientific methods such as the low-cost sensors, particularly in regions where data gaps persist. Such frameworks could help bridge the disparity in rainfall data availability between the Global North and Global South. This research should include co-design processes involving local communities, researchers and technology partners to ensure context-specific applicability and sustainability. In addition, future studies should examine the policy mechanisms and funding models that can support the implementation and scaling of these hybrid approaches.
